# The American Illustrated Dictionary

**Published:** 1907-04

**Authors:** 


**The American Illustrated Dictionary.** All the terms used in Medicine, Surgery, Dentistry, Pharmacy, Chemistry and kindred branches; with over 100 new tables. By **W. A. Newman Dorland,** M.D. Fourth Revised Edition. Octavo of 836 pages, with 293 illustrations, 119 of them in colors. Philadelphia and London: W. B. Saunders Company, 1906. (Flexible Morocco, $4.50 net; thumb indexed, $5.00 net).

This is the fourth edition of a dictionary which may be considered as wellnigh invaluable to the professional man who wishes to keep abreast of advances in medical literature, and it is just what its title page proclaims it to be, that is, revised and enlarged. There are new tables of the muscles, arteries, veins and nerves; of bacteria and bacilli; operations are classified as are symptoms, strains, tests and methods of treatment. The additions to the vocabulary of medical science are numerous and apparently complete, there being over 2,000 new words. The illustrations are excellent and prominent among the pictorial improvements are new plates of appendicitis, diphtheria, gallstones, Leishman-Donovan bodies, Koplik's sign in measles, and nephritis. The dictionary may be said to be a complete work; so thoroughly covering the ground that a recent expert witness in a more or less famous if odorous murder trial now occupying the public attention, would have escaped committing the distressful "Wileyisms" of which he was guilty had he only skimmed through the pages of this book. The matters on which he was so lamentably at sea, Romberg's sign and the consensual test, are properly explained. This we regard as a sufficient index of the completeness of this book. N. W. W.

